# Numerical Study of Using FRP and Steel Rebars in Simply Supported Prestressed Concrete Beams with External FRP Tendons

**DOI:** 10.3390/polym12122773

**Published:** 2020-11-24

**Authors:** Miao Pang, Zhangxiang Li, Tiejiong Lou

**Affiliations:** 1 Department of Civil Engineering, Zhejiang University, Hangzhou 310058, China; pm@zju.edu.cn; 2 Hubei Key Laboratory of Roadway Bridge & Structure Engineering, Wuhan University of Technology, Wuhan 430070, China; whutlizx@163.com

**Keywords:** fiber-reinforced polymer, beams, rebars, flexural strength, structural analysis

## Abstract

This study aimed at examining the feasibility of using fiber-reinforced polymer (FRP) rebars instead of steel ones in prestressed concrete beams (PCBs) with external FRP tendons. By applying an experimentally validated program, numerical tests were performed on simply supported PCBs, with investigated variables including rebars’ type and area. Three types of rebars were considered, i.e., carbon, glass FRPs (CFRP, GFRP), and reinforcing steel. The ratio of tensile rebars ranged from 0.22% to 2.16%. The results indicated that the beams with CFRP rebars exhibited better crack mode and higher ultimate load than the beams with GFRP or steel rebars. GFRP rebars led to considerably higher ultimate deflection and tendon stress increment than steel rebars. In addition, several models for calculating the ultimate stress in unbonded tendons were assessed. An analytical model was also proposed to predict the tendon stress at ultimate and flexural strength in externally PCBs with steel and FRP rebars. The model predictions agreed well with the numerical results.

## 1. Introduction

Steel corrosion would lead to the deterioration of reinforced or prestressed concrete beams (RCBs or PCBs) [1]. An effective solution to this problem is to replace steel reinforcement with fiber-reinforced polymer (FRP). In addition to their non-corrosive property, FRP composites are high-strength, light-weight, and nonmagnetic. These composite materials are increasingly used for strengthening structural elements [2,3,4,5,6,7,8]. Different types of FRP composites are available, such as aramid, basalt, carbon, and glass FRPs (AFRP, BFRP, CFRP, and GFRP). Unlike steel reinforcement with ductile characteristics, FRP composites are linear-elastic materials without yielding [9,10]. In addition, the FRP modulus of elasticity is usually lower than that of steel reinforcement [9,10]. Hence, some concerns on the use of FRP reinforcement instead of steel reinforcement may arise, e.g., ductility and deflection issues due to the lack of yielding and low modulus of elasticity for FRP composites. The bond performance of FRP reinforcement in concrete under environmental exposure is also a concern. It was generally demonstrated that harsh environments have adverse effects on FRP reinforcement’s bond durability [11,12,13].

External prestressing has been widely used in the retrofit or construction of various concrete structures. Extensive research has been conducted concerning PCBs with external FRP tendons. Grace and Abdel-Sayed [14] carried out tests on four specimens to examine the behavior of PCBs with combined externally unbonded and internally bonded CFRP tendons. Their test results showed that the ductility could be improved by strengthening using draped external CFRP tendons. Ghallab and Beeby [15] presented the test results of 16 PCBs with external AFRP tendons. They studied several factors influencing the ultimate stress in external FRP or steel tendons and proposed modifying the BS8110 equation. Abdel Aziz et al. [16] developed an analytical method to predict the load-deflection behavior of PCBs with external CFRP tendons. Wang et al. [17] tested four specimens, including 3 PCBs with external BFRP tendons and one control reinforced concrete beam (RCBs). Their study showed that using BFRP composites as external tendons for strengthening RCBs can effectively improve structural performance. Zhu et al. [18] experimentally investigated the influence of bending angle and radius on external FRP tendons’ performance. They concluded that FRP tendons’ load capacities are reduced as the bending angle increases or the bending radius decreases. Both numerical and experimental investigations [19,20] showed that behaviors of PCBs with external CFRP and steel tendons are similar.

In PCBs with external tendons, some bonded rebars need to be provided to avoid behaving as a tied arch and limit the crack width and spacing [21]. Previous studies demonstrated that externally PCBs without bonded rebars exhibit significant crack concentration, which can be effectively relieved by providing bonded rebars [19]. In existing works on PCBs with external FRP tendons, steel rebars were usually provided. The performance of conventional RCBs with FRP rebars instead of steel ones has been extensively studied [22,23,24,25,26,27,28,29,30]. However, the findings obtained from the study of RCBs with FRP/steel rebars may not be valid for PCBs with external FRP tendons as the latter is a different structural system from the former due to external prestressing. The influence of using FRP rebars instead of steel ones on the behavior of PCBs with external FRP tendons has yet to be investigated.

This paper presents a numerical and analytical study to evaluate the feasibility of providing FRP rebars instead of steel ones in PCBs with external FRP tendons. A comprehensive numerical evaluation was conducted on simply supported PCBs using an experimentally validated program, where investigated variables included the type and amount of bonded rebars. An analytical model was also proposed to predict the tendon stress at ultimate and flexural strength in externally PCBs with steel and FRP rebars.

## 2. Numerical Program and Verification

### 2.1. Numerical Program

**Figure 1:**
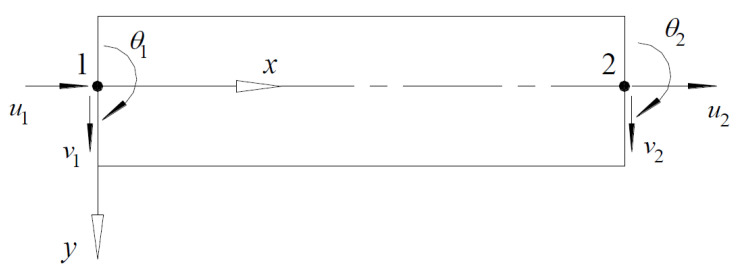
Beam element, *u*, *v*, and *θ* are the axial, transverse displacements, and rotation, respectively.

A numerical program employing the Euler–Bernoulli beam element was developed [31]. The finite element assumes negligible shear deformation, small strains with large displacements, and moderate rotations. The plane section hypothesis was also adopted. The element has six degrees of freedom as shown in Figure 1, where *u*, *v*, and *θ* are the axial, transverse displacements, and rotation, respectively, and the subscripts 1 and 2 refer to the two end nodes. A linear variation for *u* and a cubic variation for *v* along the *x*-axis is assumed. By applying an updated Lagrangian approach, the governing equations were developed, where the stiffness matrix consists of the material and geometric stiffness matrices. The effects of external tendons are transformed into equivalent loads acting on the finite elements. A detailed formulation of the numerical program can be seen in Reference [31].

**Figure 2:**
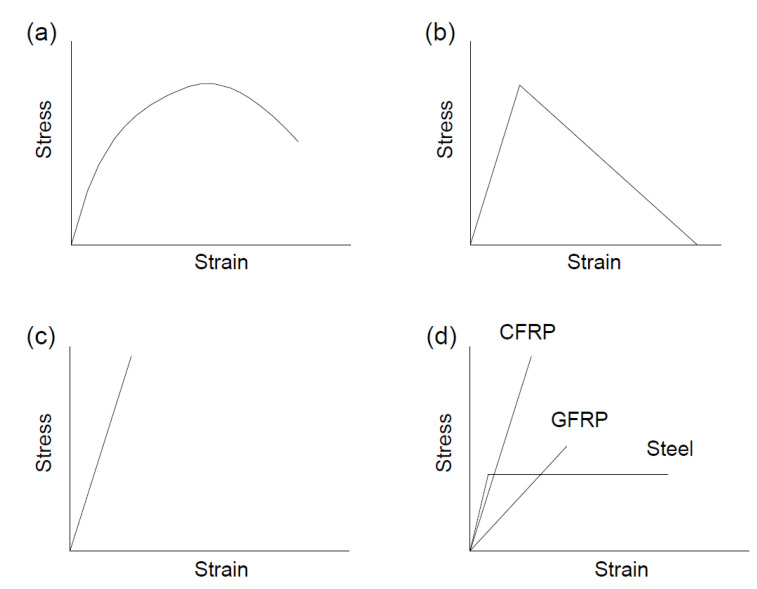
Stress–strain curves of materials. (**a**) concrete in compression recommended in Eurocode 2 [32]; (**b**) concrete in tension; (**c**) Fiber-reinforced polymer (FRP) tendons; (**d**) FRP and steel rebars.

The stress–strain relationships for materials are demonstrated schematically in Figure 2. The stress–strain relationship for concrete in compression recommended in Eurocode 2 [32] was adopted. It is shown in Figure 2a and expressed by $\sigma_{c}/f_{cm}=(k\eta -\eta^{2})/[1+(k-2)\eta ]$, where $\eta =\varepsilon_{c}/\varepsilon_{c0}$; $\sigma_{c}$ and $\varepsilon_{c}$ are the concrete stress and strain, respectively; $\varepsilon_{c0}(‰)=0.7f_{cm}^{0.31}<2.8$; $f_{cm}=f_{ck}+8$ in which $f_{ck}$ is the concrete characteristic cylinder compressive strength, in MPa; $k=1.05E_{c}\varepsilon_{c0}/f_{cm}$; and $E_{c}=22{\left(f_{cm}/10\right)}^{0.3}$, in GPa. As shown in Figure 2b, a bilinear elastic and strain-softening law is adopted for concrete in tension. FRP prestressing tendons are linearly elastic, as shown in Figure 2c. FRP rebars are also linearly elastic, while steel rebars are elastic-perfectly plastic, as shown in Figure 2d.

A typical analysis consists of two steps: load-control analysis at prestress transfer and displacement-control analysis at loading up to the ultimate. The above-mentioned numerical procedure can simulate the nonlinear behavior of PCBs with combined external FRP tendons and internal steel/FRP rebars under short-term loading up to failure.

### 2.2. Verification with Experimental Results

**Figure 3:**
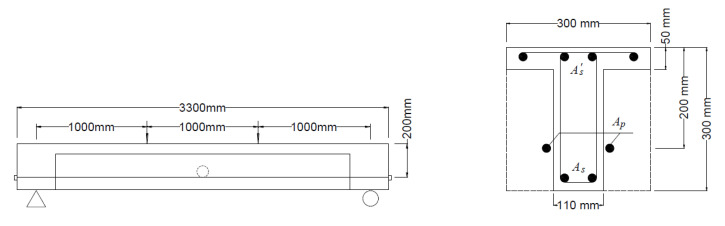
Prestressed concrete beam (PCB) specimens with external CFRP tendons [20], *A_s_*, $A_{s}^{\prime }$ and *A_p_* are the areas of tensile, compressive steel rebars and prestressing tendons, respectively.

Bennitz et al. [20] conducted a series of laboratory tests to investigate the flexural behavior of PCBs with external CFRP tendons. The main test variables were the tendon eccentricity, effective prestress, and deviator arrangement. Four of the beams (B2, B3, B6, and B7) were analyzed herein to verify the proposed model. The beams were of T-shaped section, simply supported over a span of 3.0 m, and were under third-point loading up to failure, as shown in Figure 3. The beams were post-tensioned with two horizontal straight external CFRP tendons (8 mm in diameter each) with an initial effective depth of 200 mm. Beams B2 and B3 were provided with one deviator at the midspan, while Beams B6 and B7 had no deviator. The bottom reinforcement consisted of two deformed steel rebars, each having 16 mm in diameter, while the top reinforcement consisted of four deformed steel rebars, each having 8 mm in diameter. The material parameters of the specimens are given in Table 1.

**Figure 4:**
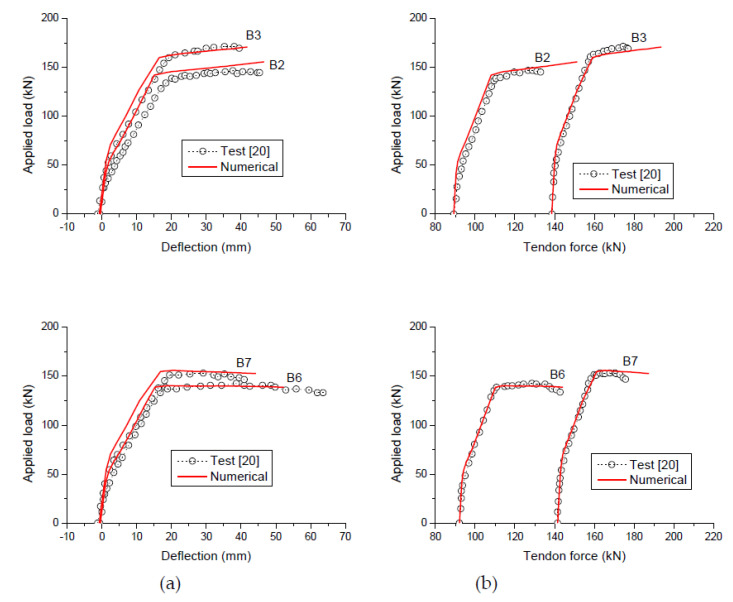
Verification with experimental results. (**a**) load versus midspan deflection; (**b**) load versus tendon force.

According to the proposed analysis, crushing failure occurs at the midspan, after significant yielding of tensile rebars (formation of a plastic hinge with sufficient rotation). This failure mode is consistent with the experimental observation. The load-displacement curves and the tendon force’s development generated by the proposed analysis are compared to the experimental data in Figure 4. It was observed that although the proposed analysis overestimated the tendon force at ultimate in some specimens (B2, B3, and B7), the numerical results were generally in satisfactory agreement with the experimental ones over the entire ranges of loading.

## 3. Numerical Evaluation

**Figure 5:**
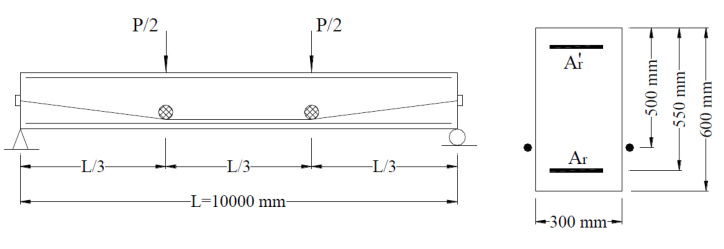
Simply supported beam for numerical investigation, *A_r_* and $A_{r}^{\prime }$ are the areas of tensile and compressive rebars, respectively.

A simply supported PCB with external tendons draped at two deviators, as shown in Figure 5, is used herein for the study. The depths of external tendons at the end supports and deviators were 300 and 500 mm, respectively. The prestressing tendons were 1000 mm^2^ in area, made of CFRP composites having the tensile strength of 1840 MPa and the modulus of elasticity of 147 GPa. The initial prestress prior to prestress transfer was 1104 MPa, namely 60% of the tensile strength. The area of tensile rebars, *A_r_*, varied from 360 to 3560 mm^2^. Thus, the value of *ρ_r_* (ratio of tensile rebars) ranged from 0.22% to 2.16%, where *ρ_r_* = *A_r_*/(*bd_r_*) in which *b* is the cross-sectional width; and *d_r_* is the depth of tensile rebars. The area of compressive rebars was 360 mm^2^. Three types of bonded rebars were considered, namely, steel, CFRP, and GFRP. The mechanical properties of the rebars are given in Table 2. The concrete strength *f_ck_* is 60 MPa.

### 3.1. Failure and Cracking Modes

**Figure 6:**
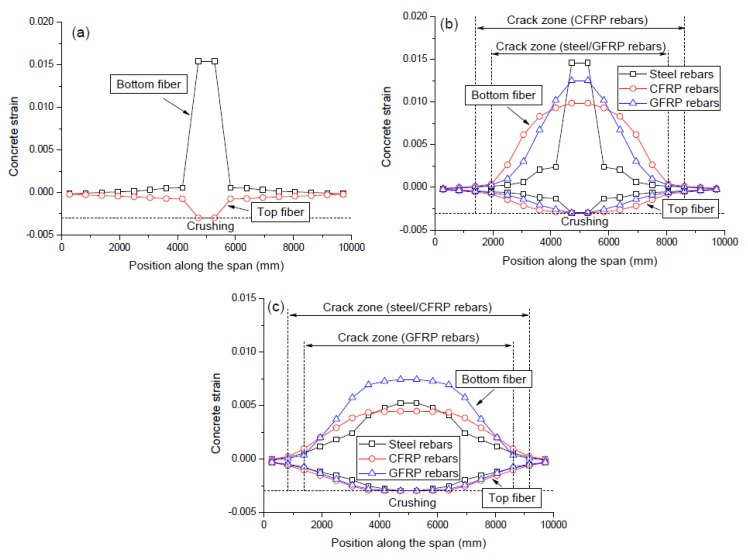
Concrete strain distribution at ultimate. (**a**) *ρ_r_* = 0.0%; (**b**) *ρ_r_* = 0.22%; (**c**) *ρ_r_* = 2.16%.

Figure 6 shows the ultimate strain distribution in the top and bottom fibers of the beams with different *ρ_r_* levels. The failure and cracking modes can be seen in the graphs of this figure. Failure of all the investigated beams occurs due to concrete crushing at midspan when the specified ultimate compressive strain of 0.003 is reached. For a beam with no rebars (*ρ_r_* = 0.0%), the noncritical sections were still far below their ultimate strain capacity at failure. When a minimum content of tensile rebars was provided (*ρ_r_* = 0.22%), the exploitation of noncritical sections was improved. Such improvement is pronounced for FRP (especially CFRP) rebars and relatively not so notable for steel rebars. However, at a high *ρ_r_* level of 2.16%, the exploitation of noncritical sections for steel rebars is nearly comparable to that for FRP rebars. At failure, both FRP tendons and rebars did not reach their rupture strength while the tensile steel rebars had yielded.

Cracks of concrete occur once the tensile strain reaches its cracking strain. Over the cracking zone, the crack width may be represented by the ultimate tensile strain. In the case for *ρ_r_* = 0%, there appears a huge tensile strain at the midspan against marginal ones over the other zones. This indicates an unfavorable crack mode, i.e., the beam has only one large crack at the midspan, and the concrete over other zones is nearly uncracked. By providing a minimum content of tensile rebars (*ρ_r_* = 0.22%), the crack mode was substantially improved, i.e., the crack width at the midspan was reduced, and more cracks occurred at the noncritical zones. The use of CFRP rebars is more effective than the use of GFRP or steel rebars to improve the crack mode. At a high *ρ_r_* level of 2.16%, the crack modes for the beams with CFRP and steel rebars are similar, while GFRP rebars lead to smaller crack zone and larger crack width over the flexural span than steel rebars.

### 3.2. Tendon Stress Development

**Figure 7:**
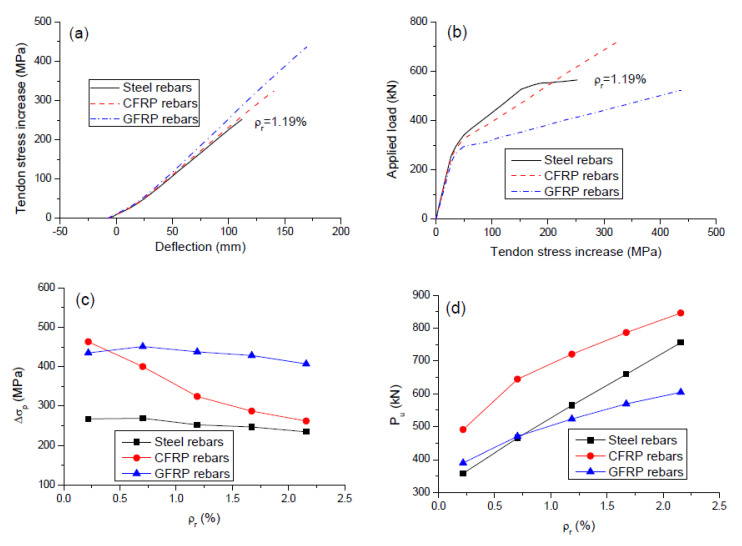
Tendon stress development for the beams with different types of rebars. (**a**) midspan deflection versus tendon stress increment; (**b**) load versus tendon stress increase; (**c**) variation of ultimate tendon stress increment with varying *ρ_r_*; (**d**) variation of ultimate load with varying *ρ_r._*

Figure 7a shows the stress increase in external tendons versus midspan deflection curves for the beams with different types of rebars (*ρ_r_* = 1.19%). There was a roughly linear relationship between the tendon stress and the deflection. The slopes for the beams with steel, CFRP, and GFRP rebars were 2.12, 2.2, and 2.46 MPa/mm, respectively. Figure 7b shows the stress increase in external tendons with the applied load. The beams with FRP rebars exhibited bilinear behavior with a turning point due to concrete cracking, while the beam with steel rebars exhibit trilinear behavior with turning points due to concrete cracking and steel yielding, respectively. Since the elastic behavior is dominated by concrete, the type of rebars appears to have practically no influence on the tendon stress evolution in the elastic range of loading. Beyond cracking, GFRP rebars develop substantially lower rebar stress because of the smaller elastic modulus and, therefore, a higher stress increase in external tendons is required at a given load level to satisfy the force equilibrium when compared to CFRP or steel rebars.

The variations of the ultimate stress increment in external tendons (Δ*σ_p_*) and the ultimate load (*P_u_*) with the *ρ_r_* level are illustrated in Figure 7c,d, respectively. As *ρ_r_* increases, the value of Δ*σ_p_* quickly decreases for the beams with CFRP rebars while it decreases slightly for the beams with GFRP or steel rebars. As expected, GFRP rebars resulted in substantially higher (around 70% higher) Δ*σ_p_* than steel rebars. At *ρ_r_* = 0.22%, the Δ*σ_p_* ratio between the beams with CFRP and steel rebars was as high as 1.73, attributed to the fact that CFRP rebars led to much better exploitation of noncritical sections than steel rebars. This ratio was reduced to 1.12 at *ρ_r_* = 2.16%. This could be explained by the comparable exploitation of noncritical sections of the beams with CFRP and steel rebars, as mentioned in the previous section. The ultimate load is dependent upon the ultimate stresses in external tendons and tensile rebars. Due to higher reinforcement stresses at ultimate, CFRP rebars led to substantially higher (37.1% higher) ultimate load than steel rebars at *ρ_r_* = 0.22%. The difference tends to decrease with increasing *ρ_r_* due to the reduced difference between reinforcement stresses in the beams with CFRP and steel rebars. At a low *ρ_r_* level of 0.22%, GFRP rebars lead to a slightly higher ultimate load than steel rebars. As *ρ_r_* increases, the increase in ultimate load for the beams with GFRP rebars is slower than that for the beams with steel rebars. As a result, when *ρ_r_* increases to a level greater than 0.77%, the ultimate load for the beams with GFRP rebars turns to be lower than that for the beams with steel rebars.

### 3.3. Deformation Behavior

**Figure 8:**
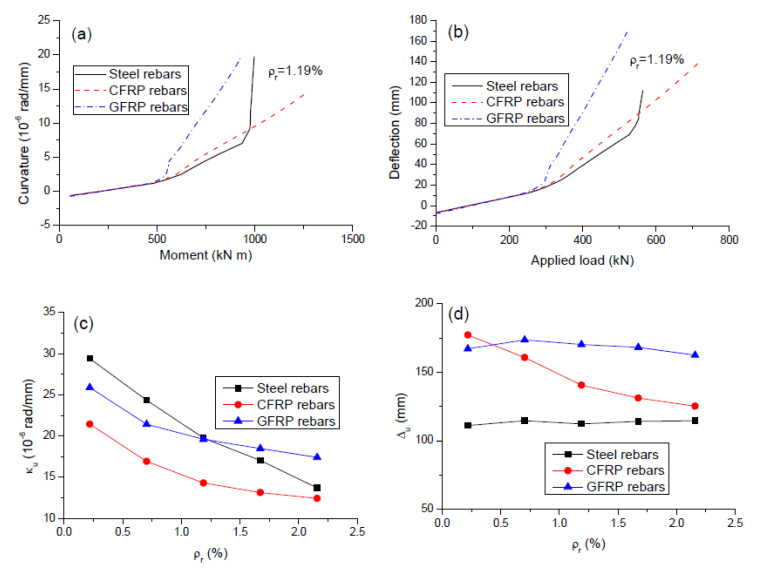
Deformation behavior for the beams with different types of rebars. (**a**) moment versus curvature at midspan; (**b**) load versus midspan deflection; (**c**) variation of ultimate curvature with varying *ρ_r_*; (**d**) variation of ultimate deflection with varying *ρ_r._*

The moment-curvature and load-deflection curves for the beams with different types of rebars (*ρ_r_* = 1.19%) are shown in Figure 8a,b, respectively. In the precracking stage, the effect of rebars on the response characteristics is negligible due to slight stress increments in rebars. The responses for the beams with FRP and steel rebars differ after cracking because the rebar contribution becomes increasingly important. The reduction in flexural stiffness due to cracking is highly dependent on the rebar modulus of elasticity, i.e., the higher the rebar modulus of elasticity, the less the reduction in member stiffness. Therefore, at a given load level, GFRP rebars lead to higher post cracking deformation than CFRP or steel rebars. The post cracking deformation for the beams with FRP rebars develops linearly until failure. For the beam with steel rebars, a significant further reduction in flexural stiffness occurs on steel yielding.

Figure 8c,d illustrates the variation of ultimate midspan curvature (*κ_u_*) and deflection (Δ*_u_*) with varying *ρ_r_*, respectively. It is seen in Figure 8c that the value of *κ_u_* decreases as *ρ_r_* increases. GFRP rebars mobilize a smaller *κ_u_* at low *ρ_r_* levels whereas a larger *κ_u_* at high *ρ_r_* levels in comparison with steel rebars. CFRP rebars mobilize lower *κ_u_* than steel rebars; the difference is substantial at low *ρ_r_* levels but reduced with increasing *ρ_r_*. It was noted that the above observation was similar to the effect of rebars on the crack width (represented by the ultimate concrete tensile strain), as discussed previously. This was because the ultimate curvature was directly proportional to the ultimate concrete tensile strain in terms of the plane section hypothesis. As shown in Figure 8d, GFRP rebars register substantially higher Δ*_u_* than steel rebars because of a significantly lower modulus of elasticity. CFRP rebars register considerably higher Δ*_u_* than steel rebars at low *ρ_r_* levels, while the difference is reduced with increasing *ρ_r_*. This could also be attributed to the rebar effect on the exploitation of noncritical sections, as explained previously.

### 3.4. Neutral Axis Depth and Rebar Strain

**Figure 9:**
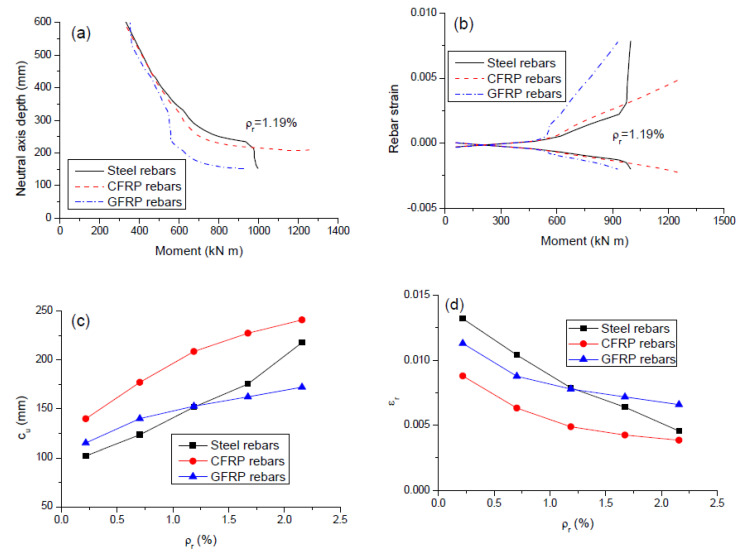
Neutral axis depth and bar strain for different types of rebars. (**a**) moment versus neutral axis depth at midspan; (**b**) moment versus rebar strain at midspan; (**c**) variation of ultimate neutral axis depth with varying *ρ_r_*; (**d**) variation of ultimate tensile rebar strain with varying *ρ_r._*

Since the neutral axis depth and rebar strain are key parameters describing flexural ductility [33], it is important to understand their behavior well. The movement of the neutral axis, after it rises to the midspan section’s bottom fiber, with the moment for the beams with different types of rebars (*ρ_r_* = 1.19%) is illustrated in Figure 9a. For the beams with FRP rebars, the neutral axis shifts rapidly with the increasing moment, and then the shift gradually slows down. Similar behavior was observed for the beam with steel rebars until yielding. After that, a fast movement of the neutral axis is resumed due to steel yielding. Figure 9b shows the strain development in FRP and steel rebars with the bending moment (*ρ_r_* = 1.19%). The behavior for the beam with steel rebars exhibited three stages with transitions caused by cracking and yielding, respectively. Two-stage behavior was observed for the beams with FRP rebars because of the lack of yielding. Due to a lower modulus of elasticity, GFRP rebars exhibited a significantly faster increase in strain after cracking but a slower stress development when compared to steel or CFRP rebars.

Figure 9c,d shows the variation of neutral axis depth (*c_u_*) and tensile bar strain at ultimate (*ε_r_*) with varying *ρ_r_*, respectively. Comparing these graphs to the graph of Figure 8c, it is seen that the effect of rebars on *κ_u_* is opposite to that on *c_u_* while coincident to that on *ε_r_*. Their theoretical relationships can explain this observation. According to the plane section hypothesis, *c_u_* and *ε_r_* are related to *κ_u_* by: $c_{u}=\varepsilon_{u}/\kappa_{u}$; $\varepsilon_{r}=\kappa_{u}(d_{r}-c_{u})=\kappa_{u}d_{r}-\varepsilon_{u}$, where *d_r_* is the depth of tensile rebars, equal to 550 mm; *ε_u_* is the ultimate concrete compressive strain, equal to 0.003. Because of a smaller value of *κ_u_*, CFRP rebars mobilize a higher *c_u_* value and a lower *ε_r_* value than steel rebars. Likewise, the values of *c_u_* and *ε_r_* mobilized by GFRP rebars could be higher or lower than those by steel rebars, depending on the *ρ_r_* level.

## 4. Analytical Modeling

### 4.1. Existing Models Using Combined Reinforcing Index for Prediction of Ultimate Stress in Unbonded Tendons

Owing to strain incompatibility, the stress in external or unbonded tendons is member-dependent. The quantification of the tendon stress is a key task in design practice. The combined reinforcing index is considered one of the best parameters used for calculating the tendon stress, as this parameter involves several important factors, including the tendon area and depth, effective prestress, rebar area, and concrete strength. The combined reinforcing index for the beams with steel rebars is defined by

(1) $$\omega_{0}=\frac{A_{p}\sigma_{pe}+A_{r}f_{y}}{bd_{p}f_{ck}}$$

where *d_p_* is the tendon depth. At ultimate, FRP rebars often reach a stress level far below their rupture strength. Therefore, the combined reinforcing index for the beams with FRP rebars could be expressed by

(2) $$\omega_{0}=\frac{A_{p}\sigma_{pe}+A_{r}\sigma_{r}}{bd_{p}f_{ck}}$$

where *σ_r_* is the stress in tensile rebars at ultimate.

**Figure 10:**
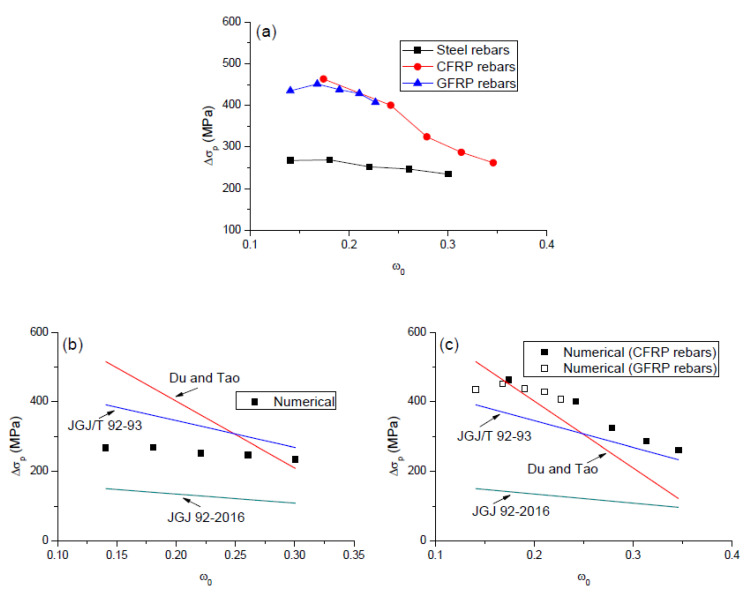
Relationship between tendon stress increment and combined reinforcing index. (**a**) numerical results; (**b**) comparison between simplified models and numerical data for the beams with steel rebars; (**c**) comparison between simplified models and numerical data for the beams with FRP rebars.

Figure 10a shows the numerical results regarding the relationship between Δ*σ_p_* and *ω*_0_ for the beams with FRP and steel rebars. It is interesting to note that the variations of Δ*σ_p_* with varying *ω*_0_ for the beams with CFRP and GFRP rebars appear to be consistent. Since CFRP represents the highest modulus of elasticity and GFRP the lowest one amongst FRP groups, it can be concluded that the beams with different types of FRP rebars follow approximately the same Δ*σ_p_*-*ω*_0_ response. At a given *ω*_0_ value, the beams with FRP rebars led to substantially higher tendon stress than the beams with steel rebars. The tendon stress tends to decrease with increasing *ω*_0_, while the decrease for the beams with FRP rebars is much quicker than that for the beams with steel rebars.

By performing laboratory tests of 22 PCBs with unbonded tendons, Du and Tao [34] found an approximately linear relationship between the tendon stress and the combined reinforcing index. They recommended the following expression for calculating Δ*σ_p_*:

(3) $$\Delta \sigma_{p}=786-1920\omega_{0}$$

JGJ/T 92-93 [35] suggested the following Equation for the computation of Δ*σ_p_*:

(4) $$\Delta \sigma_{p}=500-770\omega_{0}$$

for *L*/*d_p_* ≤ 35; and

(5) $$\Delta \sigma_{p}=250-380\omega_{0}$$

for *L*/*d_p_* > 35.

JGJ 92-2016 [36] proposed a modification of the above Equation, which is expressed as follows:

(6) $$\Delta \sigma_{p}=(240-335\omega_{0})(0.45+5.5h/L_{0})\frac{L_{2}}{L_{1}}$$

where *ω*_0_ is not greater than 0.4; *h* is the cross-sectional depth; *L*_0_ is the span length; *L*_1_ is the tendon length between end anchorages for continuous beams, and *L*_2_ is the total length of loaded spans.

It should be noted that the above models were developed for the beams with steel rebars. To evaluate the applicability of the models, the predictions by the simplified equations against the numerical data for the beams with steel rebars are presented in Figure 10b, while those for the beams with FRP rebars are presented in Figure 10c. It was observed that, in general, the Du and Tao model and JGJ/T 92-93 are unsafe, while JGJ 92-2016 is conservative when predicting the tendon stress in the beams with steel rebars. In addition, the Du and Tao model substantially overestimates the influence of *ω*_0_. For the beams with FRP rebars, the effect of *ω*_0_ on the tendon stress is overestimated by the Du and Tao model while underestimated by JGJ/T 92-93 and JGJ 92-2016. Moreover, JGJ 92-2016 appears to be overly conservative.

### 4.2. Proposed Model

**Figure 11:**
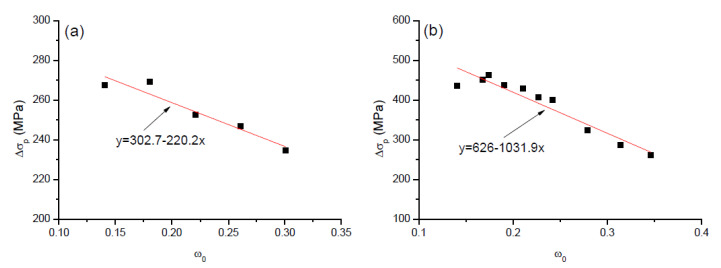
Linear fit to the numerical data. (**a**) beams with steel rebars; (**b**) beams with FRP rebars.

According to the linear fit to the numerical data of the beams with steel and FRP rebars as illustrated in Figure 11, the following Equation is proposed for predicting the stress increment in external tendons at ultimate:

(7) $$\Delta \sigma_{p}=303-220\omega_{0}$$

for the beams with steel rebars; and

(8) $$\Delta \sigma_{p}=626-1032\omega_{0}$$

for the beams with FRP rebars.

It is worth mentioning that at given cross-sectional and material properties, the value of *ω*_0_ in the beams with steel rebars is known (see Equation (1)), whereas that in the beams FRP rebars is unknown (see Equation (2)). Consequently, the value of Δ*σ_p_* in the beams with steel rebars can be calculated directly by using Equation (7), while the computation of Δ*σ_p_* in the beams with FRP rebars needs to combine Equation (8) with the section equilibrium equation.

The axial equilibrium equation of the critical section of the beams with steel rebars is

(9) $$0.85f_{ck}b\beta_{1}c_{u}=A_{p}(\sigma_{pe}+\Delta \sigma_{p})+A_{r}f_{y}-A_{r}^{\prime }f_{y}^{\prime }$$

where *β*_1_ is the stress-block factor for concrete, taken equal to 0.85; *A_r_* and $A_{r}^{\prime }$ are the area of tensile and compressive rebars, respectively. Substituting Equation (7) into Equation (9) results in

(10) $$c_{u}=\frac{A_{p}(\sigma_{pe}+303-220\omega_{0})+A_{r}f_{y}-A_{r}^{\prime }f_{y}^{\prime }}{0.85f_{ck}b\beta_{1}}$$

Hence, the flexural strength for the beams with steel rebars is calculated from

(11) $$M_{u}=A_{p}(\sigma_{pe}+\Delta \sigma_{p})d_{e}+A_{r}f_{y}d_{r}-A_{r}^{\prime }f_{y}^{\prime }d_{r}^{\prime }-0.85f_{ck}b{\left(\beta_{1}c_{u}\right)}^{2}/2$$

where *d_r_* and $d_{r}^{\prime }$ are the effective depth of tensile and compressive rebars, respectively; *d_e_* is the effective depth at ultimate of external tendons. The value of *d_e_* can be obtained by

(12) $$d_{e}=R_{d}d_{p}$$

where *R_d_* is the depth reduction factor as a result of second-order effects of externally PCBs, which may be calculated from

(13) $$R_{d}=1.14-0.005(L/d_{p})-0.19(S_{d}/L)\leq 1.0$$

for center-point loading; and

(14) $$R_{d}=1.25-0.01(L/d_{p})-0.38(S_{d}/L)\leq 1.0$$

for third-point loading.

On the other hand, the axial equilibrium of the critical section of the beams with FRP rebars is given by

(15) $$0.85f_{ck}b\beta_{1}c_{u}=A_{p}(\sigma_{pe}+\Delta \sigma_{p})+A_{r}\sigma_{r}-A_{r}^{\prime }\sigma_{r}^{\prime }$$

where

(16) $$\sigma_{r}=E_{f}\varepsilon_{u}\left(\frac{d_{r}}{c_{u}}-1\right)$$

(17) $$\sigma_{r}^{\prime }=E_{f}^{\prime }\varepsilon_{u}\left(1-\frac{d_{r}^{\prime }}{c_{u}}\right)$$

where *E_f_* and $E_{f}^{\prime }$ are the elastic modulus of tensile and compressive FRP rebars, respectively; *ε_u_* is taken equal to 0.003. Combining Equations (2), (8), (16), and (17) with Equation (15) leads to

(18) $$c_{u}=\frac{-B+\sqrt{B^{2}-4AC}}{2A}$$

where

$$A=0.85f_{ck}b\beta_{1}\;B=A_{r}E_{f}\varepsilon_{u}(1-1032\rho_{p}/f_{ck})+A_{r}^{\prime }E_{f}^{\prime }\varepsilon_{u}-A_{p}(\sigma_{pe}+626-1032\omega_{p})\;C=-A_{r}E_{f}\varepsilon_{u}d_{r}(1-1032\rho_{p}/f_{ck})-A_{r}^{\prime }E_{f}^{\prime }\varepsilon_{u}d_{r}^{\prime }$$

where

(19) $$\rho_{p}=\frac{A_{p}}{bd_{p}}$$

(20) $$\omega_{p}=\frac{A_{p}\sigma_{pe}}{bd_{p}f_{ck}}$$

Hence, the flexural strength for the beams with FRP rebars is obtained by

(21) $$M_{u}=A_{p}(\sigma_{pe}+\Delta \sigma_{p})d_{e}+A_{r}\sigma_{r}d_{r}-A_{r}^{\prime }\sigma_{r}^{\prime }d_{r}^{\prime }-0.85f_{ck}b{\left(\beta_{1}c_{u}\right)}^{2}/2$$

**Figure 12:**
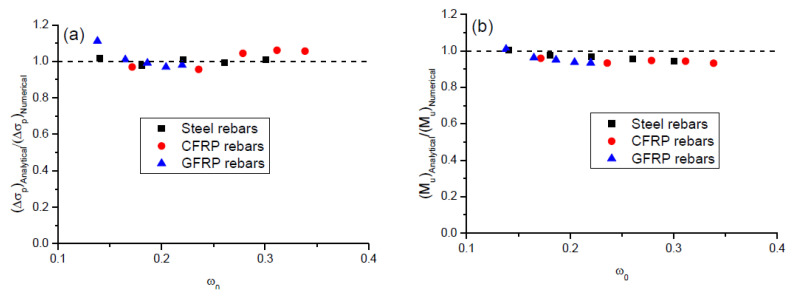
Comparison between analytical predictions and numerical results. (**a**) tendon stress increment at ultimate; (**b**) ultimate moment.

A comparison of the ultimate tendon stress increment and flexural strength predicted by the proposed analytical model with the numerical results for the beams with steel and FRP rebars is presented in Table 3 and Figure 12. It is seen that there is a good agreement between the analytical predictions and numerical data. The mean discrepancy for the ultimate tendon stress increment is 1.03% with a standard deviation of 4.08%, while that for the ultimate moment is −4.33% with a standard deviation of 2.32%.

## 5. Conclusions

A numerical and analytical study was conducted on simply supported PCBs with external CFRP tendons, aimed at identifying the effect of providing FRP bonded rebars instead of steel ones. Based on the results of the study, the following conclusions can be drawn:

- FRP (especially CFRP) rebars lead to better exploitation of noncritical sections than steel rebars, particularly notable at a low *ρ_r_* level. The crack mode is improved by providing a minimum amount of rebars, while the improvement is more effective using CFRP rebars than using GFRP or steel rebars.
- CFRP rebars lead to larger ultimate load and neutral axis depth but smaller ultimate curvature and tensile rebar strain than steel rebars. Such values registered by GFRP rebars could be larger or smaller than those by steel rebars, depending on the *ρ_r_* level.
- GFRP rebars mobilize substantially higher ultimate deflection and tendon stress increment than steel rebars. CFRP rebars lead to similar observation at a low *ρ_r_* level, while the difference between the values for the beams with CFRP and steel rebars diminishes as *ρ_r_* increases.
- Both JGJ/T 92-93 and JGJ 92-2016 underestimated the influence of combined reinforcing index on external tendons’ stress at ultimate in the beams with FRP rebars. Moreover, JGJ 92-2016 appears to be overly conservative for predicting the ultimate tendon stress.
- An analytical model was proposed to predict the tendon stress at ultimate and flexural strength in externally PCBs with steel and FRP rebars. The model predictions are in good agreement with the numerical results.

The present study is limited to simply supported conditions. Further studies on the use of FRP rebars instead of steel ones in continuous PCBs with external tendons shall be performed in the future.

## Figures and Tables

**Table 1:** Material parameters for specimens [20].

Beam	Steel Rebars						FRP Tendons				Concrete
	*A_s_* (mm^2^)	*f_y_* (MPa)	*E_s_* (GPa)	$A_{s}^{\prime }$ (mm^2^)	$f_{y}^{\prime }$ (MPa)	$E_{s}^{\prime }$ (GPa)	*A_p_* (mm^2^)	*E_f_* (GPa)	*f_f_* (MPa)	*σ_pe_* (MPa)	*f_ck_* (MPa)
B2	402	560	172	201	510	187	100.5	158	2790	895	35.2
B3	402	560	172	201	510	187	100.5	158	2790	1382	33.4
B6	402	560	172	201	510	187	100.5	158	2790	917	40.6
B7	402	560	172	201	510	187	100.5	158	2790	1407	35.9

Note: *A_s_*, *f_y_* and *E_s_* = area, yield strength, and elastic modulus of tensile steel rebars, respectively; $A_{s}^{\prime }$, $f_{y}^{\prime }$ and $E_{s}^{\prime }$ = area, yield strength, and elastic modulus of compressive steel rebars, respectively; *A_p_* = tendon area; *E_f_* and *f_f_* = elastic modulus and tensile strength of FRP composites, respectively; *σ_pe_* = effective prestress.

**Table 2:** Mechanical properties of rebars.

Rebars	Tensile Strength (MPa)	Yield Strength (MPa)	Elastic Modulus (GPa)
Steel	450	450	200
CFRP	1840	-	147
GFRP	750	-	40

**Table 3:** Comparison of ultimate tendon stress increment and moment predicted by the analytical model with numerical results.

Rebars	*ρ_r_* (%)	Δ*σ_p_* (MPa)			*M_u_* (kN m)		
		Analytical	Numerical	Error (%)	Analytical	Numerical	Error (%)
Steel	0.22	272.05	267.60	1.66	654.40	653.12	0.20
	0.70	263.25	269.05	−2.15	812.72	831.63	−2.27
	1.19	254.45	252.44	0.80	962.98	997.08	−3.42
	1.67	245.65	246.93	−0.52	1105.17	1154.67	−4.29
	2.16	236.85	234.63	0.95	1239.31	1315.42	−5.79
CFRP	0.22	448.74	463.02	−3.09	837.89	874.32	−4.17
	0.70	382.53	400.19	−4.41	1054.40	1129.98	−6.69
	1.19	338.41	324.23	4.37	1189.31	1256.83	−5.37
	1.67	304.47	287.09	6.05	1288.01	1366.46	−5.74
	2.16	276.65	261.86	5.65	1365.57	1466.04	−6.85
GFRP	0.22	483.51	435.02	11.15	713.43	706.31	1.01
	0.70	455.67	451.08	1.02	808.85	840.51	−3.77
	1.19	433.69	437.72	−0.92	882.11	928.20	−4.97
	1.67	415.19	428.42	−3.09	942.27	1004.81	−6.22
	2.16	399.09	407.03	−1.95	993.58	1063.79	−6.60
